# Influence of Rootstock on Yield Quantity and Quality, Contents of Biologically Active Compounds and Antioxidant Activity in Regent Grapevine Fruit

**DOI:** 10.3390/molecules27072065

**Published:** 2022-03-23

**Authors:** Kamila Klimek, Magdalena Kapłan, Agnieszka Najda

**Affiliations:** 1 Department of Applied Mathematics and Informatics, University of Life Science, 28 Głęboka Street, 20-612 Lublin, Poland; kamila.klimek@up.lublin.pl; 2 Institute of Horticulture Production, University of Life Science, 28 Głęboka Street, 20-612 Lublin, Poland; 3 Department of Vegetable and Herbal Crops, University of Life Sciences in Lublin, 50A Doświadczalna Street, 20-280 Lublin, Poland; agnieszka.najda@up.lublin.pl

**Keywords:** biologically active compounds, antioxidant activity, yield quantity and quality, rootstock

## Abstract

The cultivation of vines in temperate climates poses many difficulties to be overcome. The soil and climatic conditions in Poland limit the choice of vine varieties that can be used in the field; therefore, growers are often limited to varieties that are tolerant to extreme winter temperatures and spring frosts and to cultivars that are able to achieve optimum berry maturity at the end of the season. The study evaluated the effect of six rootstock types and own-root bushes on yield quantity and quality and on the content of biologically active compounds and antioxidant activity in Regent grapevine fruit. The research was conducted in 2015 at NOBILIS Vineyard (50°39′ N; 21°34′ E) in the Sandomierz Upland. Among the evaluated rootstocks, 125AA turned out to exert the significantly best effect on the yield, grape and berry weight, and number of grapes per bush. The fruit from bushes grafted on the 5BB rootstock were characterised by the highest content of L-ascorbic acid and tannins.

## 1. Introduction

Grapevine has been cultivated since ancient times and used for consumption and religious purposes [1]. In the 19th century, plantations in Europe were almost completely destroyed by phylloxera [2]. A solution to the problem was the use of rootstocks from the genus *Vitis* sp. A rootstock is the root system of a phylloxera-resistant vine, on which a scion of a selected variety is grafted [3]. Currently, this is still the most effective way to reduce phylloxera [4]. In “phylloxera-free” areas, rootstocks are used to improve crop quality and reduce the impact of adverse soil and climatic conditions [5].

The cultivation of vines in temperate climates is associated with many difficulties to be overcome. In Poland, the cultivation of this species is a relatively new branch of horticulture, and growers have to optimise agrotechnical measures in order to achieve high-quality grapes at the end of the season. The risk of vine damage by temperature drops, short growing seasons, and unfavourable soil conditions, including often too fertile and moist soils, are just a few examples of factors that affect vine cultivation and limit the choice of grape variety when establishing a vineyard [6,7]. The soil and climatic conditions found in Poland limit the choice of vine varieties that can be planted in the ground; therefore, growers are often limited to varieties that are tolerant to extreme winter temperatures and spring frosts and to cultivars that are able to achieve optimal berry maturity at the end of the season [6,7]. Although the genetic determinants of a cultivar determine the ultimate degree of cold hardiness, the environment, cultivation practices, and protection from diseases and pests influence the success of the crop.

Grafting allows grape growers to have some control over important agronomic traits and provides flexibility in growing a particular scion or variety in different soil and environmental conditions [8].

Rootstocks allow growers to plant varieties that are more adaptable and productive in specific soil and climatic conditions, and improvement of scion and rootstock combinations optimises their adaptation. Although some cold-tolerant hybrids have been grafted and tested, this is not a common practice for hybrid varieties, as rootstocks are usually used for *Vitis vinifera*. In recent years, studies have been conducted in the northern regions of the northern hemisphere to evaluate the benefits of using rootstocks for hybrid grape varieties grown in temperate and cold climates [5,9,10,11,12]. Some studies have noted that rootstocks can affect the frost resistance of scions through a faster period of acclimatisation to cold [5,12,13]. In other studies, no differences in frost hardiness were observed depending on the rootstock in many scion/rootstock combinations [14,15]. Rootstocks can also affect vine vigour, as it is the root system that provides the plant with the uptake of water and minerals necessary for growth and harbours most of the nutrient reserves that are stored over winter [10]. Some rootstocks support the physiological development of the vine and can ensure optimal ripening of the grafted variety [5,10]; hence, rootstocks also have an impact on fruit yield and quality [16,17]. Studies have shown that there is a significant interaction between grapevine cultivars and rootstocks with respect to yields, sugar accumulation in berries, berry chemistry, and flavours [5,11]. There are also papers covering different aspects of rootstock effects on grapevines, including those related to physiology [18,19], biochemistry [20,21], mineral nutrition [22,23], water deficit or excess [24,25], salinity [26], fungal diseases [27,28], viruses [29], and nematodes [30]. Others have observed more variable results, where grafted plants are often similar to those with their own root system [9]. The use of rootstocks has become a compulsory practice in commercial grapevine plantations, mainly because it provides resistance to root pests, adaptation to different soil types, and an increase in vine productivity and grape quality [31,32,33].

Grapes are among the most commonly consumed fruits in the world, both in fresh and processed form. Moreover, they have high contents of phenolic compounds (Manach et al., 2005) and a high bioactive potential, as they exhibit antioxidant, anti-inflammatory, anticancer, and antimicrobial properties [34]. These health benefits have been linked to some groups of polyphenolic compounds, such as flavonoids, phenolic acids, and stilbenes. The most important flavonoids are anthocyanins, flavonols, flavones, flavanones [35,36], catechins, epicatechins, and procyanidins [37,38]. The health benefits of polyphenols depend on the amounts consumed and their bioavailability [39].

Grapes are considered a major source of phenolic compounds, compared to other fruits and vegetables, but there is a wide variety of cultivars with different levels of these compounds in grapes [40,41]. In grapes and their by-products, phenolic compounds are directly related to sensory attributes such as colour and flavour.

The composition and phenolic properties of grapes intended for wine and juice production have been and are being continuously studied, and there is an increasing amount of such research [42,43]. Most of the data available in the literature on phenolic compounds in grapes and wines come from European countries traditionally producing wines, where *Vitis vinifera* vines are most often selected for cultivation [44]. In the case of American varieties and hybrids, which are increasingly being planted in cooler regions, there is little information on this subject [45].

Phenolic compounds are one of the main factors responsible for the antioxidant activity of fruits and vegetables. The concentration of these compounds varies depending on many factors, e.g., species, cultivars, climatic conditions, geographical region, and agrochemical treatments performed during vine growth and development [44,46,47,48,49]. Studies on the effect of rootstocks on phenolic compound content and antioxidant activity in grapes grown for wine production are scarce. As reported by Koundouras [48] and Mijowska and Ochmian [50], these parameters may depend on differences in vigour [51] and [52].

In practice, rootstocks are recommended based on environmental conditions and cultivar compatibility, which directly affects the yield and some chemical properties of the fruit, i.e., pH, acidity, and content of soluble solids. However, the nutrient content, phenolic compound concentration, and anthocyanin content are quality parameters that should be taken into account when growing grapes, mainly to ensure better selection of the most effective variety and rootstock combination [53].

Currently, the interest in grapevine cultivation in Poland is growing, and new vineyards are being established with new varieties resistant to temperature drops and major diseases such as powdery mildew and downy mildew. The grape variety Regent, which is the subject of this study, was bred in 1967 and is characterized by a dark red fruit skin [54].

In the present study, the effect of six types of rootstocks and own-root shrubs on the yield quantity and quality as well as the content of biologically active compounds and antioxidant activity of Regent grapevine fruit in Polish climate conditions was evaluated. These results can be used by growers, allotment holders, nurserymen, and scientists to develop wine industry in temperate climates.

## 2. Results and Discussion

### 2.1. Description of the Field Trial

The number of grapes per Regent grapevine bush varied from 13.8 to 22.9 and differed significantly depending on the type of rootstock used (Table 1). The statistical analysis showed the highest number of grapes in Regent vines grafted on the SO4 (22.9) rootstock and the lowest number in those grafted on the 161-49 (13.8) rootstock. There were no significant differences in the examined parameter between the own-root vines and those grafted on SO4 and 125AA, between the own-root vines and those grafted on SORI and 101-14, between those grafted on 5BB and SORI, and between the 161-49 and 5BB variants. In a study conducted by Vedoato et al. (2020), there was no significant effect of rootstocks on the number of grapes in the Niagara Rosada cultivar. Additionally, in studies conducted in eastern Canada by Provost et al. [6] and in Chile by Verdugo-Vásquez [55], there was no significant effect of the rootstock type and rootstock vines on the number of grapes per vine in cultivars Frontenac, Frontenac blanc, Marquette, and Syrah. Similar results were obtained by [56].

The fruit weight per bush ranged from 1.1 to 2.9 kg per bush, i.e., from 5.3 to 14.8 t × ha^−1^. The significantly best yielding bushes were those grafted on the 125 AA (2.9 kg) rootstock, whereas the significantly worst yields were achieved on 161-49 (1.1 kg) and 5BB (1.0 kg). As reported by Provost et al. [6], the effect of the rootstock on yields is, to a large extent, modified by the cultivar. In a study conducted in Brazil by Vedoato [57], there was no significant effect of rootstocks on the yield of the dessert grape variety Niagara Rosada, whose average yields ranged from 7.78 to 10.68 t × ha^−1^. These values were lower than those obtained by Tecchio [58] in Votuporanga (northwestern part of the São Paulo state). In the same variety grafted on IAC 766 and IAC 571-6 rootstocks, the average yields were 28.8 and 19.6 t × ha^−1^, respectively. The differences in the yields of the same variety can be justified by the different vine management systems. In the eastern region of the São Paulo state, Tecchio [58] reported that Niagara Rosada vines grafted on the IAC 572 rootstock exhibited higher productivity compared to the IAC 313 rootstock. Mota [53] also observed that the IAC 572 rootstock provided higher productivity of Niagara Rosada compared to 106–8 Mgt (Riparia do Traviú), IAC 313, and IAC 766, with mean values of 25.5, 23.8, 19.0, and 16.2 t × ha^−1^, respectively. These differences may be related to the different interactions that may occur between the cultivation method and the rootstock and soil. Kaplan [9], Harris [11], and Striegler [14] showed a significant effect of the rootstock on the yield in Regent, Norton, and Seyval grapevine cultivars, where higher yields were obtained by vines grafted on Kober 5BB, 125AA, and 110R, respectively. Reynolds and Wardle [5] evaluated the effect of four rootstocks (compared to vines with their own root system) on nine hybrid varieties in British Columbia and the northwestern United States. In all scion/rootstock combinations, the results showed a weak to moderate effect of rootstocks on the yield. A study on Cabernet Sauvignon conducted by Miele and Rizzon [22] in Brazil showed significant differences in the yield of shrubs grafted on 15 rootstock types.

The weight of one cluster ranged from 87.0 to 128.1 g and differed significantly between the evaluated combinations. The statistical analysis did not show any significant differences in the weight of clusters from bushes grafted on SO4, SORI, and 101-14 and from own-root bushes. In the study conducted by Provost et al. (2021), it was found that the weight of grapes was significantly modified by the type of rootstock used, the cultivar, and the year of research. As shown in [22], there were significant differences in cluster weight of Cabernet Sauvignon cultivar bushes grafted on 15 types of rootstocks. No significant effect of the rootstock and own-root bushes on the evaluated parameter was shown by Verdugo-Vásquez [55].

The degree of berry setting in a cluster determined by the number of berries per cluster significantly differed depending on the type of rootstock used. The self-rooted bushes and those grafted on the SO4 and SORI rootstocks were characterised by a significantly higher degree of berry filling in a cluster than in the other evaluated combinations. The bushes grafted on the 5BB rootstock formed clusters with the significantly lowest number of berries of all the evaluated plants.

The weight of one berry ranged from 1.0 to 1.9 g, the own-root bushes had the significantly smallest number of berries, and the bushes grafted on 125 AA had the significantly biggest number of berries. The weight of one berry of Frontenac, Frontenac blanc, and Marquette grapevine cultivars assessed by Provost et al. [6] significantly depended on the type of rootstock used, the cultivar, and the year of research. Irrespective of the cultivar, berries collected from the own-root bushes were the biggest among the evaluated combinations, whereas those from the 101-14 rootstock were the smallest.

The fruit extract of the Regent grapevine varied from 16.77 to 21.32 Brix and differed significantly between the evaluated combinations. It was observed that the f ruit from most of the evaluated combinations had an extract above 20 Brix, whereas the lowest value was obtained in the 101-14 variant. Similarly, Vedoato [57] showed a significant effect of the rootstock on the extract level, and this relationship was confirmed in the study conducted by Provost et al. [6]. Extract and acidity are important indicators of grape ripening and fruit quality. Higher extract content from grafted vines was observed by Reynolds and Wardle [5]. Kaplan [9] observed an effect of the rootstock on sugar content, where the lowest value was recorded for the 101-14 rootstock variant compared to others (SORI, 161-49C, 5BB, SO4, and 125AA and fruit from own-root bushes). However, in several cases, grafting had no effect on the sugar content of juice at harvest, which was found for several grape varieties [5,12].

The acidity of the fruits differed significantly from each other and depended on the type of the rootstock used. Fruits from bushes on the SO4 and 125AA rootstock had significantly higher acidity than the others. It was found that the fruits from bushes grafted on the 101-14 rootstock were characterised by the significantly lowest acidity of all the evaluated variants. In a study conducted by Vedoato [57], no significant effect of the rootstock on fruit acidity in the Niagara Rosada cultivar was shown. A similar relationship was observed in the Frontenac cultivar by Provost et al. [6], whereas significant differences between own-root bushes and those grafted on rootstocks were shown in the Frontenac blanc cultivar. In the Marquette cultivar, significant differences between rootstocks were found.

**Figure 1:**
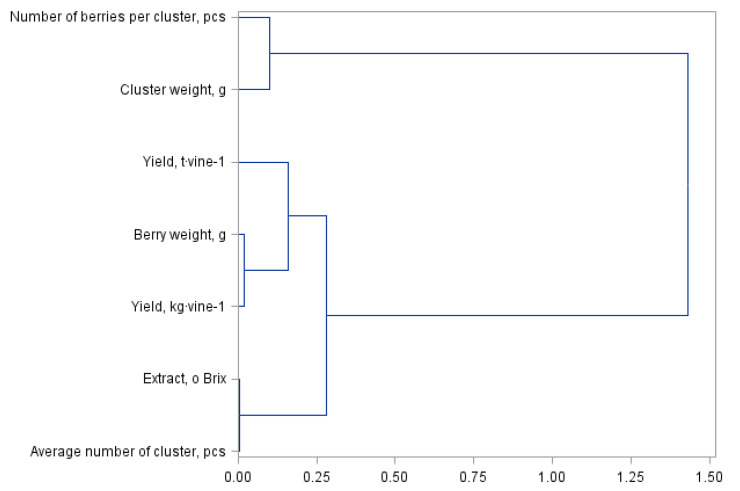
Branching-tree diagram of yield and quality parameters of self-rooted Regent variety vines.

**Figure 2:**
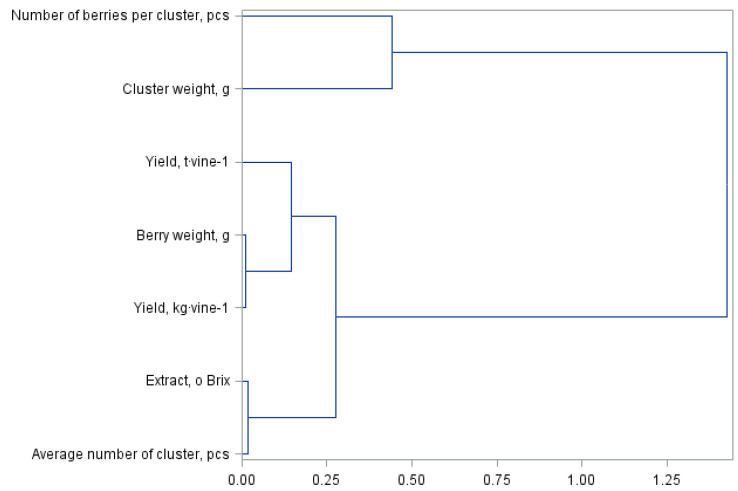
Branching-tree diagram for yield quantity and quality parameters of bushes grafted on Regent grapevine rootstocks.

Figure 1 and Figure 2 present a principal component analysis for quality and yield parameters of the Regent grapevine cultivar divided into own-root bushes and those grafted on rootstocks. The statistical analysis showed the same similarities between the assessed yield parameters; however, they formed different cluster groups. The dendrogram presented in Figure 1 forms three clusters. The first group consists of the number of berries per cluster and the cluster weight. The next cluster represents the yield per hectare, the subgroup representing the yield per bush and the weight of one berry. The last cluster is formed by the extract and the number of berries per cluster. The division in Figure 2 for shrubs grafted on the rootstocks is similar but with a difference in the number of clusters, i.e., four in this case. The first cluster shown in Figure 1 for the own-root shrubs has been divided into two separate clusters in Figure 2, which were formed from one cluster. The other clusters are the same in both analysed figures.

### 2.2. Description of Biologically Active Compounds

The content of L-ascorbic acid in the fruits of the studied grapevine cultivars ranged from 27.3 to 45.7 mg × 100 g^−1^ and was significantly modified by the type of rootstock used. Fruits from bushes grown on rootstock 5BB (45.7 mg × 100g^−1^) were characterised by the highest vitamin C content, whereas its lowest content was detected in those from rootstocks SO4 (27.3 mg × 100 g^−1^), 161-49 (28.0 mg×100 g^−1^), and 101-14 (28.3 mg×100 g^−1^).

The content of phenolic acids in the Regent grapevine fruit was significantly modified by the type of rootstock used. The SO4 rootstock had a significant positive effect on the content of the assessed parameter (Table 2). Fruits coming from bushes grafted on 101-14 had the lowest content of phenolic acids (0.13 mg × 100 g^−1^). There were no significant differences in the level of the analysed parameter between fruits from the bushes grafted on 125AA, 5BB, and SORI and the own-root variant. Mijowska et al. (2017) evaluated the cultivar Regent in cool climate conditions and showed the lowest amount of phenolic acids in grapes growing on the Börner rootstock (6.63 mg × 100 g^−1^ FW), whereas other plants were characterised by significantly higher content of these compounds in fruit (7.9–10.4 mg × 100 g^−1^ FW). As demonstrated by Yang et al. [59], Anastasiadi et al. [60], and Bunea et al. [61], the quantitative and qualitative composition of phenolic acids is significantly influenced by genetic features, environmental conditions, and agrotechnical management.

Total flavonoids ranged from 0.07 to 0.11 and showed a significant difference between the combinations, with the highest level in the fruit from the 125AA grafted vines. Significant differences in total flavonoids can be attributed to several factors, e.g., genetic factors, climate, vineyard management, grape ripening level, berry size, and extraction method [42]. Flavonoids are the best cofactors for anthocyanin pigmentation in wine. The higher the flavonoid content in grapes, the higher the amount of anthocyanins transferred to the wine during the winemaking process (Schwarz et al., 2005). A study conducted by da Silva [43] showed a significant effect of the rootstock on the flavonoid sum in only two varieties, Merlot and IAC 138-22 Máximo; no such relationship was shown in the other cultivars. Mijowska et al. [62] found the highest level of flavonols in grapevine fruits of the Regent cultivar collected from plants grafted on Sori (38.04 mg × 100 g^−1^ FW) and Kober 125AA (34.11 mg × 100 g^−1^ FW) rootstocks. Grapes grown on their own roots or on other types of rootstocks (25.84–27.01 mg × 100 g^−1^ FW) were characterised by low flavonol content. In a study conducted by Satish et al. [63], the flavonoid content of Thompson Seedless grapes was higher in grapes grafted on rootstocks compared to non-grafted grapes.

The anthocyanin content in the Regent grapevine fruit was significantly modified by the type of rootstock used. It was shown that fruits from the own-root bushes (713.5 mg × 100 g^−1^ FM) were characterised by the significantly highest anthocyanin content, whereas the lowest content was determined in fruits from the bushes grafted on SORI (331.4 mg × 100 g^−1^ FM) and 101-14 (389.9 mg × 100 g^−1^ FM). In a study conducted in Brazil [43], the anthocyanin content in red grapes was significantly different among cultivars, whereas no significant effect of the rootstock on the evaluated parameter was observed in the case of Isabel, Bordô, Merlot and Syrah cultivars. As shown in the case of the Cabernet Sauvignon cultivar analysed by Da Silva [43], differences in the total anthocyanin content between rootstocks vary depending on the region, whereas the rootstocks used in experiments are very often different, and the results are probably related to the intrinsic characteristics of these plants, vigour, and environmental conditions at the harvest site. In a study conducted by Mijowska [62], the highest anthocyanin content was found in grapes from plants grafted on SORI and Kober 125AA rootstocks (423 and 400 mg × 100 g^−1^ FW, respectively), and the lowest level was determined in fruit from plants with their own roots and on Börner and Kober 5BB rootstocks (350, 355, 360 mg × 100 g^−1^ FW, respectively). In turn, Suriano [64] observed the highest levels of anthocyanins in berries of the Greco Nero cultivar grafted on 775 Paulsen and Kober 5BB rootstocks. In a study reported by Ehrhardt [54], berries of the Regent cultivar grown in Germany and Italy had anthocyanin content of 120–130 mg × 100 g^−1^ FW, respectively. Several factors can influence the content of this pigment in grapes, e.g., species, cultivar, ripening stage, climatic conditions at the growing site [65], and the rootstock used. Many studies indicate that anthocyanins present in grapes are concentrated mainly in the skin [45,66]. Therefore, the anthocyanin values obtained in the present work are lower because the whole berry (skin, pulp, and stone) was used for analysis, thus diluting the anthocyanin values by the pulp. Pozzan et al. [41] demonstrated the effect of different rootstocks on the anthocyanin content of Bordo grapes. As shown by these authors, the highest content of these compounds was detected in Bordô grapes grafted on the IAC 766 versus 106 rootstock. Numerous studies have demonstrated that the anthocyanin and tannin contents largely depend on the cultivar, species, fruit ripeness degree, production localization, and climate [61,67,68,69].

The tannin content ranged from 0.14 to 0.30%. Fruits from the bushes grafted on 5BB were characterised by the highest tannin content, whereas those in the SO4 variant had the significantly lowest value of this parameter. As demonstrated by Matthews and Nuzzo [70], tannins are present in the skin, seeds, and peduncles. Their content in fruit juice (must) and wine depends on the crop technique, shrub loading, climatic conditions, methods of maceration, and fermentation circumstances. These compounds have a spectrum of important properties that affect the colour, colour stability, astringency, and wine depth [71]. Numerous studies have shown that the anthocyanin and tannin content is highly dependent on the cultivar, species, fruit ripeness, production site, and climate [59,67,68,69].

The antioxidant activity of the Regent grapevine fruit extracts significantly depended on the type of rootstock used, and DPPH ranged from 56.4 to 87.0 µM TE × g^−1^ FM. Fruits from the shrubs grafted on SORI were characterised by the significantly highest antioxidant activity, whereas the lowest value of this parameter was determined in the 5BB variant. Several literature studies of antioxidant activity reported difficulties in obtaining similar data for grapes. This makes it difficult to compare the data due to such factors as the use of different analytical methods (e.g., DPPH, ABTS, FRAP, ORAC), standards and units of measurement, or even differences in the grape reference material [43]. However, it is known that the antioxidant activity of grapes is correlated with the content of phenolic compounds [42,47,72].

**Figure 3:**
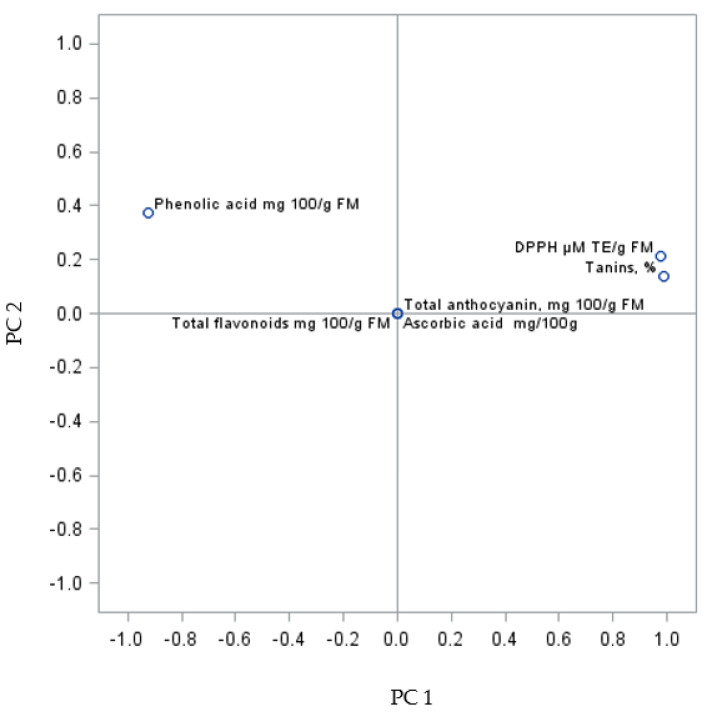
PCA analysis of biologically active compounds in Regent grapevine fruit from own-root bushes.

The PC sum for the analysed fruits was 100.00% (PC1 93.13% and PC2 6.87%) (Figure 3). The analysis of the biologically active compounds and antioxidant activity of fruits from the self-rooted bushes showed three clusters. The first group consists of phenolic acids, the second one comprises antioxidant activity and tannins, and the third largest cluster consists of total flavonoids, anthocyanin content, and L-ascorbic acid.

**Figure 4:**
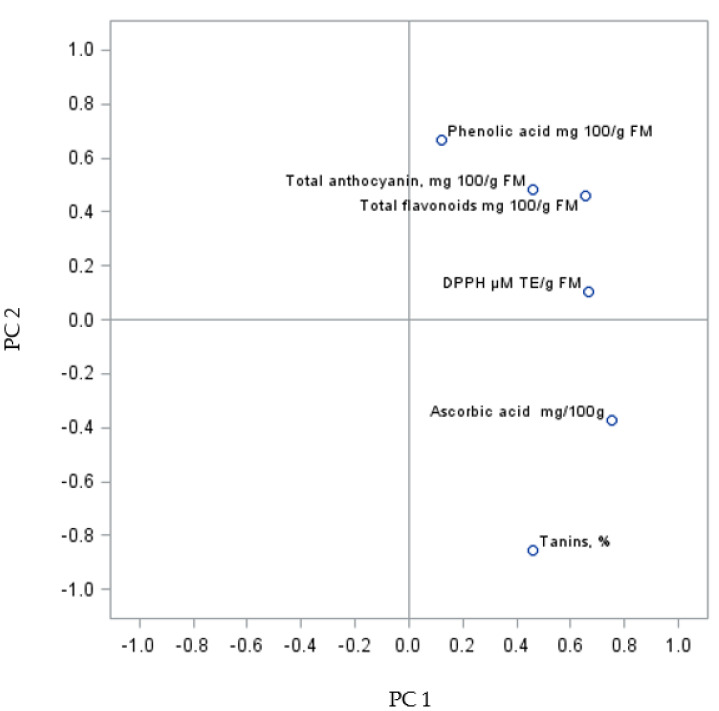
PCA analysis of biologically active compounds in Regent grapevine fruit from shrubs improved on rootstocks.

The PC sum for the analysed grape fruits of the Regent variety was 60.83% (PC1 31.31% and PC2 29.52%) (Figure 4). The analysis of the biologically active compounds and antioxidant activity of the fruits revealed a similarity between some parameters forming two groups. The first relationship was found between the content of phenolic acids, the sum of anthocyanins and flavonoids, and the DPPH parameters. The second relationship was shown between vitamin C content and tannins. Da Silva et al. [43] showed a positive correlation between antioxidant activity and total anthocyanins, flavonoids, and phenolic compounds in red grapes, which is in agreement with results reported by other authors [42,47,72]. However, there are also several other studies showing no correlation or a negative correlation between these variables [73], implying that the variation is related to different cultivars, rootstocks, and growing sites.

### 2.3. Analysis of the Relationship between Yield Parameters and Biologically Active Compounds

The multivariate Pearson correlation analysis showed a strong positive correlation between total fruit acidity and phenolic acids, as an increase in one of these parameters was accompanied by an increase in the other one (Table 3). A positive correlation was observed between the extract and total anthocyanins, between the number of berries per cluster and the DPPH level, and between the weight per cluster and berry weight and total flavonoids. An increase in the yield size and quality parameters was associated with an increase in the correlated chemical composition parameters.

## 3. Materials and Methods

### Plant Materials

The research was conducted in 2015 at NOBILIS Vineyard (50°39′ N; 21°34′ E) in the Sandomierz Upland, southeastern Poland. The research material consisted of Regent variety vines, which were planted in spring 2010 at 2.0 × 1.0 m spacing (5000 pcs × ha^−1^) on lessive soil made from loess, which comprised 2.1% of organic matter. The plants were managed as a single Guyot twine with a trunk height of 40 cm, a single bed of about 0.9 m length, and 1 two-eyed pivot. In the course of the experiment, regular protection against diseases, pests, and weeds was provided in accordance with the current vine protection recommendations. The bushes were not irrigated, and soil pH ranged from 6.0 to 6.5, depending on the study year. In the bud-bursting phase, Hydrocomplex fertilizer (12N-11P-18K) was applied in the soil at a dose of 300 kg ha^−1^, and other macro- and microelements were supplemented through foliar fertilization when needed.

In the field experiment, the influence of the rootstock type on the size and quality of the Regent grapevine yield was assessed. The vines of the studied cultivars grew on 6 types of rootstocks: 125 AA, SORI, 101-14, SO4, 5BB, and 161-49. Non-grafted bushes growing on their own roots were the control.

The following parameters were analysed: number and weight of clusters, number and weight of berries, and total extract content. The yield and number of grapes per bush were determined by counting and weighing berries from each bush with an accuracy of 0.001 kg. Average berry weight was determined by weighing and counting berries from five medium-sized clusters from each replicate. The experiment was set up in a randomized block design and included 7 combinations with 5 repetitions. The repetitions were plots in which 3 plants were growing.

The analytical evaluation was carried out in the Laboratory for Vegetable and Herbal Material Quality at the Department of Vegetable Crops and Medicinal Plants, University of Life Sciences in Lublin. All reagents and solvents were analytical grade chemicals from Merck (Darmstadt, Germany), Sigma Chemical Co. (St. Louis, MO, USA), or POCh (Gliwice, Poland).

### Physicochemical Analyses

The fruit extract content was measured on the harvest day using a refractometer Abbe WAY 2W (EnviSense, Lublin, Poland) in juice squeezed from 100 representative berries collected from each combination. In order to determine biologically active compounds and antioxidant activity, the grapes were transported to the laboratory on the harvest day, stored in a cooler at 8 °C for 16 h, and finally, subjected to chemical analyses. Titratable acidity (TA) was determined in accordance with Polish Norm PN-90/A/75101/02.

### Determination of L-Ascorbic Acid

Fresh comminuted grape fruits (5 g) were extracted twice for 30 min with 2.5 mL 4.0% (m/V) L-cysteine and 10.0 mL of water by sonification. All aqueous extracts were combined and diluted with water to 25 mL. The samples were analysed using high-performance liquid chromatography. The analyses were performed using a LaChrom-Merck HPLC system with a photodiode array detector (DAD L-7450), and all separations were carried out in a Lichrospher 100 RP18 column (250.0 × 4.0 mm, 5.0 μm; Merck). The mobile phase consisted of 0.0272 g × L KH_2_PO_4_ adjusted to pH 2.40, with H_3_PO_4_ applied in isocratic elution for 30 min. The flow rate was adjusted to 1.0 mL/min. The detection wavelength was set to DAD at λ = 254.0 nm. All 20.0 μL samples were injected. All separations were performed at 24.0 °C. The peaks were assigned by spiking the samples with standard compounds and comparing the UV spectra and retention times (ascorbic acid, 5.66 min) [55]. Calibration curves were obtained from five doses of each external standard (0.01 to 1.40 mg/mL). The regression coefficient (R2) of the calibration curve for ascorbic acid was equal to Y = 85.231, X = 18.787. The RSD value for the repeatability (*n* = 4) of the standard solution was 0.40% (0.01 mg/mL ascorbic acid). The limits of quantitation (LOQ) and detection (LOD) of ascorbic acid were 0.16 and 0.04 mg/L, respectively. All solvents used were HPLC-grade Merck (Darmstadt, Germany). Reference standards were obtained from Sigma Chemical Co. (St. Louis, MO, USA) and POCH (Gliwice, Polska).

Total phenolic acid estimation was carried out according to the Arnov method (Polish Pharmacopoeia, 2002). In total, 1 mL of sample was mixed with 5 mL of distilled water, 1 mL 0.5 M HCl, 1 mL of Arnov reagent, and 1 mL 1 M NaOH and subsequently adjusted to 10 mL with distilled water. Absorbance was measured at 490 nm. The total phenolic acid content was expressed as caffeic acid equivalent (CAE).

### Estimation of Anthocyanins by Means of Colorimetry

Samples of raw material (1.0 g) were extracted with 50 mL HCl (1 mol × dm) and heated in a water bath for 1 h. The extract was hydrolysed with 20 mL *n*-butanol, and then two 10 mL *n*-butanol portions were added as a solution. The anthocyanin extracts were rinsed with *n*-butanol in a 50 mL flask. Absorbance was measured immediately at 533 nm [56].

The percentage of anthocyanins expressed as delphinidin chloride was calculated from the equation:

(1) $$P=\frac{A\times V\times F}{M}$$

where: P = total anthocyanins (mg × 100 g), A = absorbance at 533 nm, V = value of the butanol phase (50 mL), F = coefficient expressed as delphinidin chloride (2.6), and m = mass of sample to be examined (mg).

### Determination of Antioxidant Activity (AA)

A 0.1 mL aliquot of the methanol extract prepared as described above was mixed with 3.9 mL of an 80% ethanolic 0.6 mM DPPH solution. The tubes were vortexed for 15 s and allowed to stand for 180 min, as described by Cai et al. (2003). Afterwards, the absorbance of the mixture was measured at λ = 517 nm wavelength using a HITACHI UV-Vis spectrophotometer (UV-Vis model U-2900′ Shimadzu, Kyoto, Japan). Most tested compounds reacted completely within 180 min in these conditions. The reaction time for vitamin C was less than 1 min due to its fast oxidation. Ethanol (80%) was used as a blank solution, and a DPPH solution without the analysed samples (3.9 mL of DPPH + 0.1 mL of 80% ethanol) served as the control. All tests were performed in triplicate. The antiradical activity of the samples was expressed as the median effective dose for radical scavenging activity (EC50): TP (mg) of the antioxidant (test sample) required for a 50% decrease in the absorbance of DPPH radicals and inhibition (%) of DPPH absorbance = (*A*_control_ − *A*_test_) × 100/*A*_control_. A plot of the absorbance of DPPH vs. the dose of the antioxidant was made to establish the standard curves (dose–response curves) and to calculate EC50. *A*_control_ is the absorbance of the control (DPPH solution without the test sample), and *A*_test_ is the absorbance of the test sample (DPPH solution plus 0.1 mL of 5 μM test compound). Ascorbic acid served as a standard. The results of the assay were expressed relative to an ascorbic acid equivalent.

### Estimation of Total Flavonoids

The studied material was investigated for the total content of flavonoids using a modified Christ and Müller method, calculated for quercetin QE Polish Pharmacopoeia [74]. Absorbance was measured at 425 nm on a HITACHI U-2900 spectrophotometer.

The content of flavonoids was calculated from the equation:

(2) $$X=\frac{8.75\times A}{m}$$

where m (g) was the amount of fresh mass

### Tannin Estimation

Tannins were determined with the Folin–Ciocalteu method. About 0.1 mL of the sample extract was added to a volumetric flask (10 mL) containing 7.5 mL of distilled water, 0.5 mL of Folin–Ciocalteu phenol reagent, and 1 mL of a 35% Na_2_CO_3_ solution, and diluted to 10 mL with distilled water. The mixture was shaken well and kept at room temperature for 30 min. A set of reference standard solutions of gallic acid (20, 40, 60, 80, and 100 μg × mL) was prepared in the same manner as described earlier. The absorbance for the test and standard solutions was measured against the blank at 725 nm with a UV/Visible spectrophotometer. The tannin content was expressed in % of GAE of the extract Polish Pharmacopoeia, 2011 [75] according to the European Pharmacopoeia [76].

## 4. Statistical Analysis

After completion of the experiment, the results were subjected to statistical analysis using one-way analysis of variance. Moreover, the results were presented in a graphical form. Inference was performed at the significance level of α = 0.05. Using Pearson correlation coefficients, correlations were established between individual grape quality parameters. In addition, multivariate data analysis techniques were performed using cluster analysis to group the rootstock types into homogeneous groups, such that the objects in one cluster were more similar to each other than to the objects in the other clusters. The results of the analysis were presented by means of a dendrogram. All analyses were performed using SAS Enterpriese Guide 5.1 software. (Copyright © SAS Institute Inc., SAS Campus Drive, Cary, North Carolina 27513, USA, accessed on 14 February 2022).

## 5. Conclusions

In *Vitis vinifera*, some studies have shown significant effects of rootstocks on the vine yield and fruit composition, but the number of studies on the effects of rootstocks on hybrid grape varieties is still very limited. The present study carried out in temperate climate conditions showed that the rootstocks significantly affected the yield size and quality parameters, the content of biologically active compounds, and the antioxidant activity of the Regent cultivar. Among the evaluated rootstocks, 125AA turned out to exert the significantly best effect on the yield, grape, and berry weight and the number of grapes per bush. The fruits from bushes grafted on the 5BB rootstock were characterised by the highest content of L-ascorbic acid and tannins. The highest contents of phenolic acids, total flavonoids, antioxidant activity, and anthocyanins were detected in the SO4, 125AA, SORI, and own-root variants, respectively.

## Figures and Tables

**Table 1:** Influence of rootstock type on the quantity and quality of the Regent grapevine yield.

Root Stock	Average Number of Cluster, pcs	Yield, kg∙vine^−1^	Yield, t∙vine^−1^	Cluster Weight, g	Number of Berries per Cluster, pcs	Berry Weight, g	Solid Content, Brix	Total Acidity, %
101-14	19.2 ± 4.87 ^B^	2.1 ± 0.83 ^B^	9.9 ± 4.56 ^B^	101.4 ± 20.21 ^B^	78.8 ± 4.48 ^B^	1.4 ± 0.329 ^BC^	16.77 ± 0.72 ^B^	0.43 ± 0.010 ^D^
125 AA	24.3 ± 5.09 ^A^	2.9 ± 0.46 ^A^	14.8 ± 2.53 ^A^	128.1 ± 9.64 ^A^	74.3 ± 31.12 ^B^	1.9 ± 0.343 ^A^	20.58 ± 0.17 ^A^	0.59 ± 0.026 ^A^
161-49	13.8 ± 7.45 ^D^	1.1 ± 0.51 ^C^	5.3 ± 2.52 ^C^	87.3 ± 13.85 ^C^	70.1 ± 13.12 ^B^	1.2 ± 0.238 ^BCD^	20.83 ± 0.09 ^A^	0.51 ± 0.006 ^B^
5 BB	15.2 ± 10.77 ^CD^	1.5 ± 1.21 ^C^	7.6 ± 5.99 ^C^	87.0 ± 29.81 ^C^	55.1 ± 10.91 ^C^	1.5 ± 0.339 ^B^	20.27 ± 0.22 ^A^	0.52 ± 0.015 ^B^
Own root	20.8 ± 3.28 ^AB^	2.2 ± 0.22 ^B^	10.9 ± 1.11 ^B^	98.1 ± 5.24 ^B^	92.2 ± 10.83 ^A^	1.0 ± 0.114 ^D^	21.32 ± 0.26 ^A^	0.48 ± 0.052 ^C^
SO4	22.9 ± 7.12 ^A^	2.4 ± 0.68 ^B^	12.1 ± 3.45 ^B^	103.6 ± 17.94 ^B^	88.1 ± 14.62 ^A^	1.3 ± 0.188 ^BCD^	19.71 ± 0.14 ^A^	0.61 ± 0.021 ^A^
SORI	18.4 ± 8.14 ^BC^	1.9 ± 0.99 ^BC^	9.6 ± 4.97 ^BC^	102.6 ± 20.64 ^B^	94.2 ± 13.14 ^A^	1.1 ± 0.267 ^CD^	20.58 ± 0.17 ^A^	0.53 ± 0.032 ^B^
*p*-value	*0.0263*	*<0.0001*	*<0.0001*	*0.0003*	*<0.0001*	*<0.0001*	*<0.0001*	*<0.0001*

Mean values marked with the same letters: A, B, C, D do not differ significantly at α = 0.05.

**Table 2:** Effect of the rootstock on the content of biologically active compounds and antioxidant activity of Regent grapevine fruit.

Rootstock	Ascorbic Acid mg 100 × g^−1^	Phenolic Acid mg 100 × g^−1^ FM	Total Flavonoids mg 100 × g^−1^ FM	Total Anthocyanin, mg 100 × g^−1^ FM	Tannins, %	DPPH µM Te × g^−1^ FM
101-14	28.3 ± 2.51 ^D^	0.13 ± 0.001 ^D^	0.08 ± 0.001 ^B^	338.9 ± 53.91 ^F^	0.21 ± 0.001 ^C^	70.7 ± 0.12 ^C^
125 AA	34.6 ± 0.57 ^C^	0.18 ± 0.001 ^B^	0.11 ± 0.005 ^A^	529.0 ± 0.01 ^B^	0.17 ± 0.001 ^D^	81.3 ± 0.12 ^B^
161-49	28.0 ± 1.01 ^D^	0.15 ± 0.000 ^C^	0.07 ± 0.001 ^C^	396.3 ± 0.01 ^E^	0.21 ± 0.001 ^C^	60.7 ± 0.12 ^D^
5 BB	45.7 ± 4.04 ^A^	0.18 ± 0.000 ^B^	0.08 ± 0.007 ^B^	456.9 ± 13.61 ^C^	0.30 ± 0.001 ^A^	56.4 ± 0.12 ^E^
Own root	37.0 ± 0.01 ^C^	0.17 ± 0.014 ^B^	0.08 ± 0.000 ^B^	713.5 ± 0.01 ^A^	0.20 ± 0.001 ^C^	78.1 ± 0.12 ^B^
SO4	27.3 ± 1.53 ^D^	0.21 ± 0.032 ^A^	0.08 ± 0.001 ^B^	444.9 ± 0.01 ^D^	0.14 ± 0.001 ^E^	62.1 ± 0.12 ^D^
SORI	42.0 ± 2.64 ^B^	0.17 ± 0.000 ^B^	0.08 ± 0.001 ^B^	331.4 ± 58.81 ^F^	0.26 ± 0.001 ^B^	87.0 ± 0.12 ^A^
*p*-value	*<0.0001*	*<0.0001*	*<0.0001*	*<0.0001*	*<0.0001*	*<0.0001*

Mean values marked with the same letters: A, B, C, D, E, F do not differ significantly at α = 0.05.

**Table 3:** Correlation coefficient for yield and quality parameters as well as biologically active compounds of Regent grapevine and their significance.

	Ascorbic Acid mg × 100 g^–1^	Phenolic Acid mg × 100 g^–1^ FM	Total Flavonoids mg × 100 g^–1^ FM	Total Anthocyanin, mg 100 × g^–1^ FM	Tannins %	DPPH µM TE × g^–1^ FM
Average number of cluster, pcs	−0.1655	0.3205	0.2548	0.2054	−0.3303	0.2346
Yield, kg∙vine^–1^	−0.1316	0.3456	0.4285	0.2281	−0.3416	0.3486
Yield, t∙vine^–1^	−0.1209	0.3557	0.4388	0.2338	−0.3361	0.3487
Cluster weight, g	−0.1908	0.1340	0.5386	0.1210	−0.2719	0.4258
Number of berries per cluster, pcs	−0.2420	0.0473	0.0014	0.1006	−0.3455	0.5674
Berry weight, g	0.0001	0.1874	0.5752	0.0513	−0.0597	−0.0459
Solid content, °Brix	0.4078	0.4137	0.0413	0.5339	0.0819	0.1376
Total acidity, %	0.0039	0.7769	0.2897	0.0361	−0.4866	−0.0294

## Data Availability

Not applicable.

## Sample Availability

Samples of the compounds are available from the authors.
